# Lewy body-like alpha-synuclein inclusions trigger reactive microgliosis prior to nigral degeneration

**DOI:** 10.1186/s12974-018-1171-z

**Published:** 2018-05-01

**Authors:** Megan F. Duffy, Timothy J. Collier, Joseph R. Patterson, Christopher J. Kemp, Kelvin C. Luk, Malú G. Tansey, Katrina L. Paumier, Nicholas M. Kanaan, Luke D. Fischer, Nicole K. Polinski, Olivia L. Barth, Jacob W. Howe, Nishant N. Vaikath, Nour K. Majbour, Omar M. A. El-Agnaf, Caryl E. Sortwell

**Affiliations:** 1 Department of Translational Science and Molecular Medicine, Michigan State University, 400 Monroe Avenue NW, Grand Rapids, MI 49503-2532 USA; 2 Neuroscience Graduate Training Program, Michigan State University, Grand Rapids, MI USA; 3 MD/PhD Program, Michigan State University, Grand Rapids, MI USA; 4 Mercy Health Hauenstein Neuroscience Medical Center, Grand Rapids, MI USA; 5 Center for Neurodegenerative Disease Research, Department of Pathology and Laboratory Medicine, University of Pennsylvania Perelman School of Medicine, Philadelphia, PA USA; 6 Department of Physiology, Emory University School of Medicine, Atlanta, GA USA; 7 Neurological Disorders Research Center, Qatar Biomedical Research Institute (QBRI), Hamad Bin Khalifa University (HBKU), Education City, Qatar; 8 Life Sciences Division, College of Science and Engineering, Hamad Bin Khalifa University (HBKU), Education City, Qatar

**Keywords:** Neuroinflammation, Parkinson’s disease, Animal models, Synucleinopathy, Microglia, Major-histocompatibility complex-II, Neurodegeneration, Selective vulnerability

## Abstract

**Background:**

Converging evidence suggests a role for microglia-mediated neuroinflammation in Parkinson’s disease (PD). Animal models of PD can serve as a platform to investigate the role of neuroinflammation in degeneration in PD. However, due to features of the previously available PD models, interpretations of the role of neuroinflammation as a *contributor to* or a *consequence of* neurodegeneration have remained elusive. In the present study, we investigated the temporal relationship of neuroinflammation in a model of synucleinopathy following intrastriatal injection of pre-formed alpha-synuclein fibrils (α-syn PFFS).

**Methods:**

Male Fischer 344 rats (*N* = 114) received unilateral intrastriatal injections of α-syn PFFs, PBS, or rat serum albumin with cohorts euthanized at monthly intervals up to 6 months. Quantification of dopamine neurons, total neurons, phosphorylated α-syn (pS129) aggregates, major histocompatibility complex-II (MHC-II) antigen-presenting microglia, and ionized calcium-binding adaptor molecule-1 (Iba-1) immunoreactive microglial soma size was performed in the substantia nigra. In addition, the cortex and striatum were also examined for the presence of pS129 aggregates and MHC-II antigen-presenting microglia to compare the temporal patterns of pSyn accumulation and reactive microgliosis.

**Results:**

Intrastriatal injection of α-syn PFFs to rats resulted in widespread accumulation of phosphorylated α-syn inclusions in several areas that innervate the striatum followed by significant loss (~ 35%) of substantia nigra pars compacta dopamine neurons within 5–6 months. The peak magnitudes of α-syn inclusion formation, MHC-II expression, and reactive microglial morphology were all observed in the SN 2 months following injection and 3 months prior to nigral dopamine neuron loss. Surprisingly, MHC-II immunoreactivity in α-syn PFF injected rats was relatively limited during the later interval of degeneration. Moreover, we observed a significant correlation between substantia nigra pSyn inclusion load and number of microglia expressing MHC-II. In addition, we observed a similar relationship between α-syn inclusion load and number of microglia expressing MHC-II in cortical regions, but not in the striatum.

**Conclusions:**

Our results demonstrate that increases in microglia displaying a reactive morphology and MHC-II expression occur in the substantia nigra in close association with peak numbers of pSyn inclusions, months prior to nigral dopamine neuron degeneration, and suggest that reactive microglia may contribute to vulnerability of SNc neurons to degeneration. The rat α-syn PFF model provides an opportunity to examine the innate immune response to accumulation of pathological α-syn in the context of normal levels of endogenous α-syn and provides insight into the earliest neuroinflammatory events in PD.

**Electronic supplementary material:**

The online version of this article (10.1186/s12974-018-1171-z) contains supplementary material, which is available to authorized users.

## Background

The etiology of Parkinson’s disease (PD) is stochastic: a culmination of aging-related changes in brain environment, genetic predispositions, and environmental insults that result in accumulation of alpha-synuclein (α-syn) inclusions (i.e., Lewy bodies) and degeneration of nigrostriatal dopamine neurons [1, 2]. Converging evidence suggests a role for microglia-mediated neuroinflammation in human PD. This theory is supported by observations of increased inflammatory cytokines in both PD patient cerebral spinal fluid (CSF) and plasma [3, 4] and in the patient brain as longitudinal PET imaging has demonstrated early and sustained microglial activation in the basal ganglia [5]. Furthermore, postmortem analyses in PD patients revealed increased expression of inflammatory markers such as human leukocyte antigen (HLA-DR), major histocompatibility complex-II (MHC-II), phagocytic marker CD68, intercellular adhesion molecule-I (ICAM-1), and integrin adhesion molecule (LFA-1) in the substantia nigra [6, 7]. However, a drawback of biofluid and postmortem PD brain samples is that they only provide a static snapshot of events within a longitudinal cascade of PD pathophysiology. This is especially problematic as the overwhelming majority of PD patient samples are collected from individuals who have likely harbored PD-related pathology for decades before, if also not after, diagnosis [8]. This confounds interpretations of the role of neuroinflammation in degeneration in PD and prevents the understanding as to whether neuroinflammation participates as a contributor to nigral degeneration or is simply an artifact of cell death.

One of the most consistent observations in postmortem PD tissue is an increase in the number of microglia expressing MHC-II (HLA-DR in humans [7, 9, 10]), a cell surface protein on antigen-presenting cells which is necessary for CD4+ T cell infiltration. More recently, gene expression changes related to inflammation, including an upregulation of MHC-II, have also been noted in incidental Lewy body disease subjects (Braak stages 1–3 [10, 11]). Additionally, a variant in the HLA-DR gene which encodes for MHC-II is associated with amplified risk for development of PD after pesticide exposure [12]. Increased MHC-II is often concurrently upregulated with genes for proinflammatory cytokines such as tumor necrosis factor (TNF) and interleukin-1 beta (IL-1β) [13]. Moreover, decreased MHC-II expression was shown to attenuate downstream secretion of proinflammatory cytokines [14, 15]. Taken together, it is likely that MHC-II is most closely associated with a proinflammatory phenotype in microglia and may play a contributory role in nigral degeneration in PD. However, while the concept that MHC-II expression on microglia is increased in PD patients is not novel [9], the *temporal pattern* of observed increases in MHC-II in relation to α-syn aggregation and/or nigrostriatal degeneration has been unable to be systematically examined.

Animal models of PD can serve as platforms to investigate the role of neuroinflammation in PD-related cell death and dysfunction. The neuroinflammatory consequences of nigral degeneration and/or α-syn aggregation have been examined previously in various models, including but not limited to, neurotoxicant models (6-hydroxydopamine (6-OHDA) [16, 17]; 1-methyl-4-phenyl-1,2,3,6-tetrahydropyridine (MPTP) [18, 19]); transgenic models expressing human wild-type or mutant α-syn (A503T, A30P [20–22]) and viral vector-mediated overexpression of human wild-type or mutated α-syn in the nigrostriatal system [22–28]. However, certain characteristics of these models limit interpretations regarding the specific initiator of the neuroinflammation observed—synuclein inclusions and/or degeneration. Neurotoxicant models (6-OHDA, MPTP) rarely exhibit α-syn pathology [18, 29]. Transgenic models generally do not recapitulate marked nigrostriatal degeneration despite widespread, α-syn pathology [21, 30]. Whereas a robust inflammatory response is observed in association with the elevated α-syn levels, aggregates, and nigral degeneration in viral vector-based α-syn overexpression models [22–28, 31–33], the contribution of supraphysiological α-syn levels or the α-syn species difference (human α-syn expressed in rat or mouse) to the neuroinflammatory response is unclear. Importantly, in human sporadic PD, total α-syn levels are not increased; rather, phosphorylation and the ratio of soluble to insoluble α-syn increases over time [34–36].

**Fig. 1:**
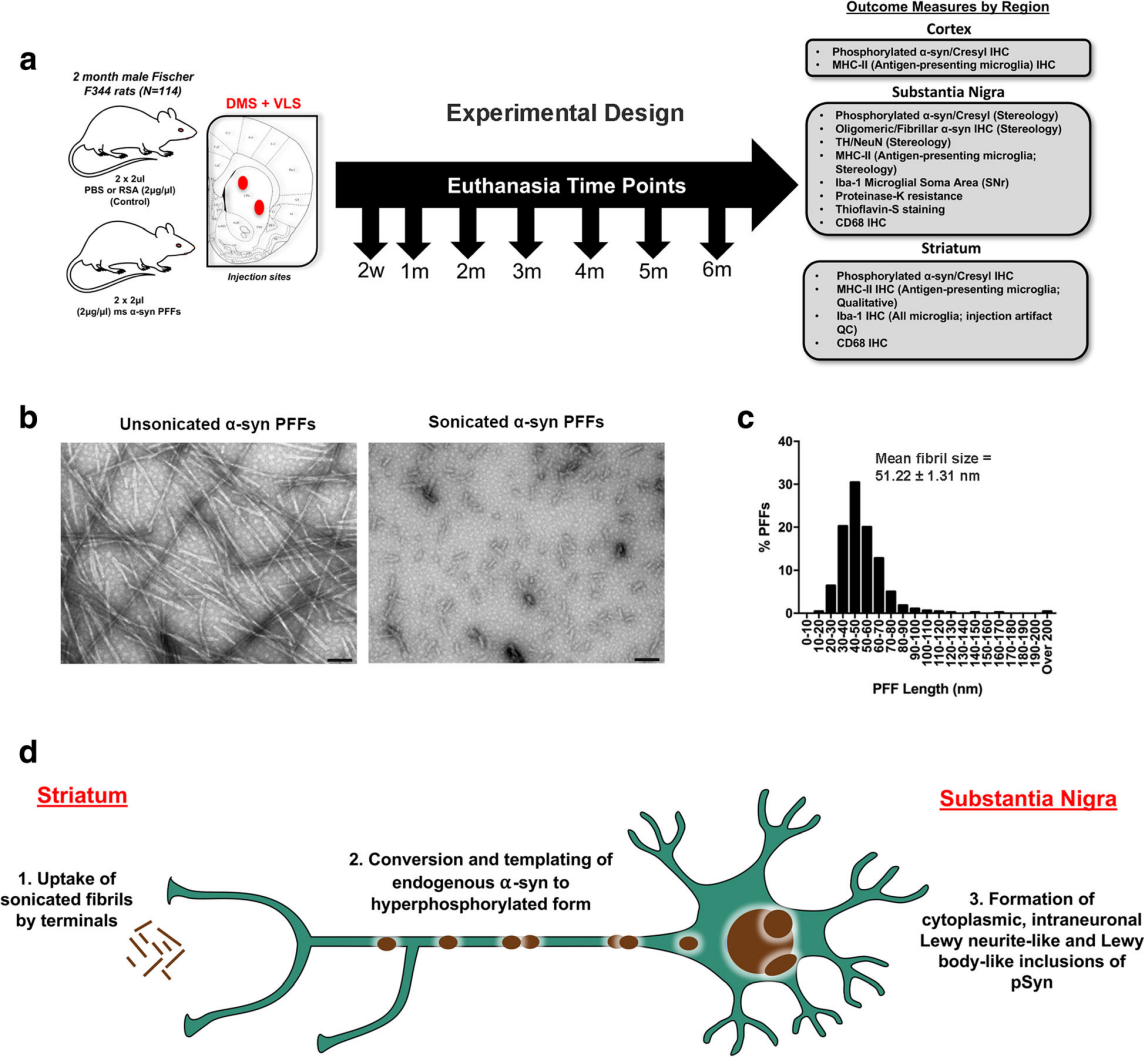
Experimental design and PFF quality control. **a** Experimental design: 2-month-old male Fischer344 rats (*N* = 114) received two unilateral intrastriatal injections of either sonicated α-syn PFFs, Dulbecco’s PBS (PBS), or rat serum albumin (RSA; follow-up study only). Cohorts of rats were euthanized at an early time point (2 weeks) and monthly intervals thereafter. Brains were removed and processed for immunohistochemical measures of pathology as detailed. **b** Electron micrographs of unsonicated (*left*) α-syn PFFs and sonicated α-syn PFFs (*right*); scale bars = 100 nm. **c** Measurement distribution of ~ 500 sonicated PFFs prior to injection; mean fibril size = 51.22 ± 1.31 nm. **d** Schematic of PFF model of synucleinopathy. Sonicated α-syn fibrils are injected into the striatum and taken up by nigrostriatal terminals (*1*), after which they template and convert endogenous α-syn to a hyperphosphorylated, pathological form (*2*), ultimately accumulating into Lewy neurite- and Lewy body-like inclusions (*3*). Abbreviations: α-syn = alpha-synuclein; PFFs = pre-formed alpha-synuclein fibrils; PBS = phosphate-buffered saline; DMS = dorsal medial striatum; VLS = ventrolateral striatum; IHC = immunohistochemistry; pSyn = α-syn phosphorylated at serine 129; MHC-II = major histocompatibility complex-II; Iba-1 = ionized calcium-binding adaptor molecule 1; TH = tyrosine hydroxylase; NeuN = neuronal nuclei; CD68 = cluster of differentiation 68; QC = quality control

An alternative model of the key features of human sporadic PD such as (1) protracted development of α-syn inclusions under conditions of (2) normal expression levels of endogenous α-syn during an interval that (3) precedes significant nigrostriatal degeneration would offer distinct advantages and allow the time course and potential impact of neuroinflammation to be delineated. Recently, our lab has characterized a rat model of PD that recapitulates this sequence of events, extending previous findings in mice [37, 38]. In this model, nigrostriatal synucleinopathy is induced by intrastriatal injection of sonicated pre-formed α-syn fibrils (α-syn PFFs) into wild-type rats [37, 39]. The fibrils act as seeds to template and trigger normal levels of *endogenous* α-syn to accumulate into misfolded hyperphosphorylated, pathological α-syn (Fig. 1a). The initial injection of α-syn PFFs per se does not directly cause toxicity, given that α-syn pathology and nigral degeneration do not occur in α-syn knockout animals injected with PFFs [38]. In this model, we observe a widespread accumulation of intraneuronal Lewy neurite-like and Lewy body-like inclusions of phosphorylated α-syn (pSyn) in areas that innervate the striatum. Importantly, the accumulation of intracellular pSyn is gradual and results in loss of striatal dopamine and metabolites in addition to ~ 40% loss of SNc dopamine neurons over 6 months [37]. Thus, the synucleinopathy produced in the α-syn PFF model provides a unique opportunity to examine the neuroinflammatory consequences of α-syn inclusion accumulation in the context of normal levels of endogenous, intracellular α-syn. In the present study, we systematically investigated the temporal profile of Lewy body-like phosphorylated α-syn inclusion load, reactive microglial morphology, MHC-II antigen presentation, and degeneration in the SN. Importantly, we observe reactive microglia and increased microglial MHC-II expression in association with peak load of SNc pSyn inclusions *months prior to degeneration*, suggesting that neuroinflammation may contribute to nigrostriatal degeneration.

## Methods

### Animals

Young adult (2 months), male Fischer344 rats (*n* = 114) were used in this study. All animals were provided food and water ad-libitum and housed at the AAALAC-approved Van Andel Research Institute vivarium. All procedures were approved and conducted in accordance with Institute for Animal Use and Care Committee (IACUC) at Michigan State University.

### Preparation of α-syn PFFs and verification of fibril size

Purification of recombinant, full-length mouse α-syn and in vitro fibril assembly was performed as previously described [39–41]. Prior to sonication, α-syn fibrils were assessed to verify lack of contamination (LAL Assay (~ 1 endotoxin units/mg), high molecular weight (sedimentation assay), beta sheet conformation (thioflavin T), and structure (electron microscopy). Prior to injection, PFFs were thawed, diluted in sterile Dulbecco’s PBS (DPBS, 2 μg/μl), and sonicated at room temperature using an ultrasonicating homogenizer (300VT; Biologics, Inc., Manassas, VA) with the pulser set at 20% and power output at 30% for 60 pulses at 1 s each. Following sonication, a sample of the PFFs was analyzed using transmission electron microscopy (TEM). Formvar/carbon-coated copper grids (EMSDIASUM, FCF300-Cu) were washed twice with ddH_2_O and floated for 1 min on a 10-μl drop of sonicated α-syn fibrils diluted 1:20 with DPBS. Grids were stained for 1 min on a drop of 2% uranyl acetate aqueous solution; excess uranyl acetate was wicked away with filter paper and allowed to dry before imaging. Grids were imaged on a JEOL JEM-1400 transmission electron microscope. The length of over 500 fibrils per sample was measured to determine average fibril size. The mean length of sonicated mouse α-syn PFFs was estimated to be 51.22 ± 1.31 nm, well within the optimal fibril length previously reported to result in seeding of endogenous phosphorylated α-syn inclusions in vitro and in vivo (Fig. 1b, c) [42].

### Intrastriatal injections

Sonicated PFFs were kept at room temperature during the duration of the surgical procedures. All rats were deeply anesthetized with isoflurane received two 2-μl unilateral intrastriatal injections (4 μl total; AP + 1.6, ML + 2.4, DV − 4.2; AP − 1.4, ML + 2.0, DV − 7.0 from the skull) either of sonicated mouse α-syn PFFs (2 μg/μl as described previously [37]) or an equal volume of DPBS at a rate at 0.5 μl/min (*n* = 6 per treatment per time point). Injections were administered made using a pulled glass needle attached to a 10-μl Hamilton syringe. After each injection, the needle was left in place for 1 min, retracted 0.5 mm, left in place for an additional 2 min, and then slowly withdrawn. Animals were monitored post-surgery and euthanized at predetermined time points (14, 30, 60, 90, 120, 150, and 180 days; Fig. 1). In a subsequent experiment, rats received two 2-μl unilateral intrastriatal injections either of mouse α-syn PFFs 2 μg/μl, DPBS, or rat serum albumin (RSA, Sigma-Aldrich, St. Louis, MO; 9048-46-8; 2 μg/μl) at the identical coordinates and were euthanized at 2 months postinjection (*n* = 6 per treatment).

### Immunohistochemistry

All animals were euthanized via pentobarbital overdose (60 mg/kg) and intracardially perfused with heparinized 0.9% saline followed by cold 4% paraformaldehyde in 0.1 M PO_4_. Brains were extracted and postfixed in 4% PFA for 48 h and placed in 30% sucrose until they sunk. For sectioning, brains were frozen on a sliding microtome and cut at 40 μm. Free-floating sections (1:6 series) were transferred to 0.1 M tris-buffered saline (TBS). Following the washes, endogenous peroxidases were quenched in 3% H_2_O_2_ for 1 h and rinsed in TBS. Sections were blocked in 10% normal goat serum/0.5% Triton X-100 in TBS (NGS, Gibco; Tx-100 Fischer Scientific) for 1 h. Following the blocking, sections were immunolabeled with primary antibodies: mouse anti-α-syn fibrils/oligomers (O2; 1:5000 [43]) or mouse anti α-syn fibrils (F2; 1:5000 [43]), pan rabbit-anti α-syn (Abcam, Cambridge, MA; AB15530, 1:1000), mouse anti-phosphorylated α-syn at serine 129 (pSyn, 81A; Abcam, Cambridge, MA; AB184674; 1:10,000), rabbit anti-tyrosine hydroxylase (TH; Millipore, Temecula, CA; MAB152, 1:4000), rabbit anti-ionized calcium-binding adaptor molecule-1 (Iba-1; Wako, Richmond, VA; 019-19741, 1:1000), mouse anti-neuronal nuclei (Neu-N; Millipore, Temecula, CA; MAB 377, 1:5000), or mouse anti-rat major histocompatibility complex-II for antigen-presenting microglia (MHC Class II RT1B clone OX-6, Bio-Rad, Hercules, CA; MCA46G, 1:5000) overnight in 1% NGS/0.5% Tx-100/TBS at 4 °C. Following the washes, sections were incubated in biotinylated secondary antibodies (1:500) against mouse (Millipore, Temecula, CA; AP124B) or rabbit IgG (Millipore, Temecula, CA; AP132B) followed by washes in TBS and 2-h incubation with Vector ABC standard detection kit (Vector Laboratories, Burlingame, CA; PK-6100). Labeling for pSyn, MHC-II, and TH was visualized by development in 0.5 mg/ml 3,3′ diaminobenzidine (DAB; Sigma-Aldrich St. Louis, MO; D5637-10G) and 0.03% H_2_O_2_. For dual brightfield visualization of Neu-N and Iba-1, sections were developed according to the manufacturer’s instructions using the Vector ImmPACT DAB Peroxidase (Vector Labs, Burlingame, CA; SK-4605) and ImmPACT VIP Peroxidase (Vector Labs, Burlingame, CA; SK-4105) kits, respectively. Slides were dehydrated in ascending ethanol series and then xylenes before coverslipping with Cytoseal (Richard-Allan Scientific, Waltham, MA). A subset of pSyn-labeled sections were also counterstained with cresyl violet for quantification of *intraneuronal* pSyn inclusions in the SNc.

### RNAscope in situ hybridization for Iba-1 and MHC-II IHC

Forty-micrometer-thick striatal tissue sections were incubated in pretreat 1 from the RNAscope Pretreatment Kit (Advanced Cell Diagnostics, Hayward, CA; 310020) for 1 h. Sections were washed in TBS and then mounted on VistaVision HistoBond slides (VWR, Randor, PA; 16004-406) and placed on slide warmer at 60 °C overnight. Slides were then incubated for 10 min in pretreat 2 at 99 °C and washed twice in water. Tissue was outlined with Pap Pen (Abcam, Cambridge, UK; ab2601), incubated with pretreat 3 in a hybridization oven at 40 °C for 15 min, washed twice in water, and incubated with the probe for AIF1 (Iba1; Advanced Cell Diagnostics, Hayward, CA; 457731) for 2 h in the hybridization oven at 40 °C. Six amplification steps with the amplification buffers (Advanced Cell Diagnostics, Hayward, CA; 320600) were then performed in alternating 30- and 15-min incubation intervals in the hybridization oven per manufacturer instructions. Tissue was developed using the supplied DAB reagent (Advanced Cell Diagnostics, Hayward, CA; 320600). Tissue was then counterstained for MHC-II (RT1B clone OX-6, Bio-Rad, Hercules, CA; MCA46G, 1:500) in a hybridization chamber, following the same procedures as detailed for other immunohistochemical stains with the exception that the Vector SG reagent (Vector Laboratories, Burlingame, CA) was used as the chromogen. Slides were rinsed in TBS and coverslipped with Cytoseal 60. Images were taken on a Nikon Eclipse 90i microscope with a QICAM camera (QImaging, Surrey, British Colombia, Canada).

### Quantification of TH, NeuN, pSyn, and MHC-II immunoreactive profiles

Microbrightfield (MBF) Stereoinvestigator (MBF Bioscience, Williston, VT) was used to estimate the total population of THir and NeuNir neurons to determine the time course of TH phenotype loss and overt nigral degeneration. Contours were drawn around the SNc using the ×4 objective on every sixth section through the rostrocaudal axis (9–10 sections). A series of counting frames (50 μm × 50 μm) was systematically and randomly distributed over grid (183 μm × 112 μm) placed over the SNc, allowing for quantification of approximately 20% of the SNc. An investigator blinded to experimental conditions counted THir and NeuNir cells using the optical fractionator probe with a ×60 oil immersion objective. Markers were placed on each THir or NeuNir cell in a 1–2-μm z-stack within the counting frame. Between 50 and 500 objects were counted to generate stereological estimates of the total cell population. The total population estimate was calculated using optical fractionator estimates, and variability within animals was assessed via the Gunderson coefficient of error (< 0.1). Due to heterogeneity in the distribution of both pSyn and MHC-II immunoreactive profiles within the SN, total enumeration rather than counting frames was used for quantification. Neurons with intraneuronal pSyn inclusions were defined as profiles of dark, densely stained pSyn immunoreactivity within cresyl violet-positive neurons. Contours were drawn around the SNc using the ×4 objective on every sixth section through the entire rostrocaudal axis of the SNc (9–10 sections). pSyn inclusions and MHC-IIir microglia were then systematically counted within each contour using the ×20 objective. Numbers represent the raw total number of pSyn inclusions or MHC-IIir microglia per animal multiplied by 6 to extrapolate the population estimate.

### Microglial soma area analysis

Forty-micrometer-thick nigral tissue sections (1:6 series) from animals injected with α-syn PFFs, RSA, or and DPBS 2 months and 6 months following injection were dual labeled for NeuN and Iba-1 as described above to distinguish the SNc from the SNr. The three nigral sections adjacent to the sections containing the most pSyn inclusions were identified. z-stack images of the ipsilateral and contralateral SNr bordering the SNc were taken on a Nikon Eclipse 90i microscope with a QICAM camera (QImaging, Surrey, British Colombia, Canada) using the ×20 objective and analyzed with Nikon Elements AR (version 4.50.00, Melville, NY). Using the auto-detect feature, each Iba-1ir soma’s border was outlined and adjusted accordingly to obtain an accurate quantification of area of the soma, excluding any processes. All microglia in the field of view of each z-stack per section, per rat were quantified with total number of microglia per rat calculated (100–250). Data are expressed as mean Iba-1ir soma area per treatment group. Soma measurements for all microglia per treatment were also grouped into 10 μm bins and expressed as a percentage of total microglia counted.

### Thioflavin-S staining

1:12 series was washed in TBS and subsequently mounted on subbed slides to dry (~ 1 h). Slides were incubated in 0.5% KMnO_4_ in TBS for 25 min, followed by five washes in TBS. Sections were destained in 0.2% K_2_S_2_O_5_/0.2% oxalic acid in TBS for 3 min followed by incubation in 0.0125% thioflavin-S in 40% EtOH/TBS for 3 min and differentiated in 50% EtOH for 15 min. Sections were rinsed first in TBS and then ddH_2_0 before coverslipping with VECTASHIELD Mounting Medium for fluorescence.

### Proteinase-K digestion

1:12 nigral series was washed in TBS. A subset of free floating tissue sections was treated with 10 μg/ml proteinase K (Invitrogen, Carlsbad, CA; 25530015) for 30 min at room temperature, followed by three washes in TBS and four washes in TBS-Tx. Sections were then processed for pan α-syn immunohistochemistry (rabbit anti-α-syn, Abcam, Cambridge, UK; AB15530) as described above, mounted on subbed slides, dehydrated to xylenes, and coverslipped.

### Statistics

Statistical analyses were performed using IBM SPSS Statistics (IBM, Armonk, NY) or GraphPad Prism (La Jolla, CA). Statistical significance for all cases was set at *p* < 0.05. Statistical outliers were assessed using the Absolute Deviation from the Median (ADAM) method using the “very conservative” criterion [44]. To compare numbers of O2 vs. F2 immunoreactive cells (Fig. 4), THir and NeuNir neurons (Fig. 5), pSyn α-syn inclusions (Fig. 6), MHC-IIir microglia (Fig. 5), and Iba-1ir microglia number and size (Fig. 7), a one-way ANOVA with Tukey’s post hoc analyses was used. Correlation analysis was conducted to investigate the relationship between ipsilateral and contralateral THir neurons (Fig. 5) and between MHC-IIir and pSyn α-syn inclusions (Fig. 6).

## Results

### Sonicated α-syn PFFs are the optimal size for pathology induction in vivo

Prior to intrastriatal injection of mouse α-syn PFFs, we investigated the size of the PFFs following sonication using transmission electron microscopy (TEM; Fig. 1b, c). The mean length of sonicated mouse α-syn PFFs was estimated to be 51.22 ± 1.31 nm, well within the optimal fibril length previously reported to result in seeding of endogenous phosphorylated α-syn inclusions (Fig. 1d, schematic) in vitro and in vivo [42].

### Unilateral intrastriatal injection of α-syn PFFs induces widespread Lewy-like pathology

**Fig. 2:**
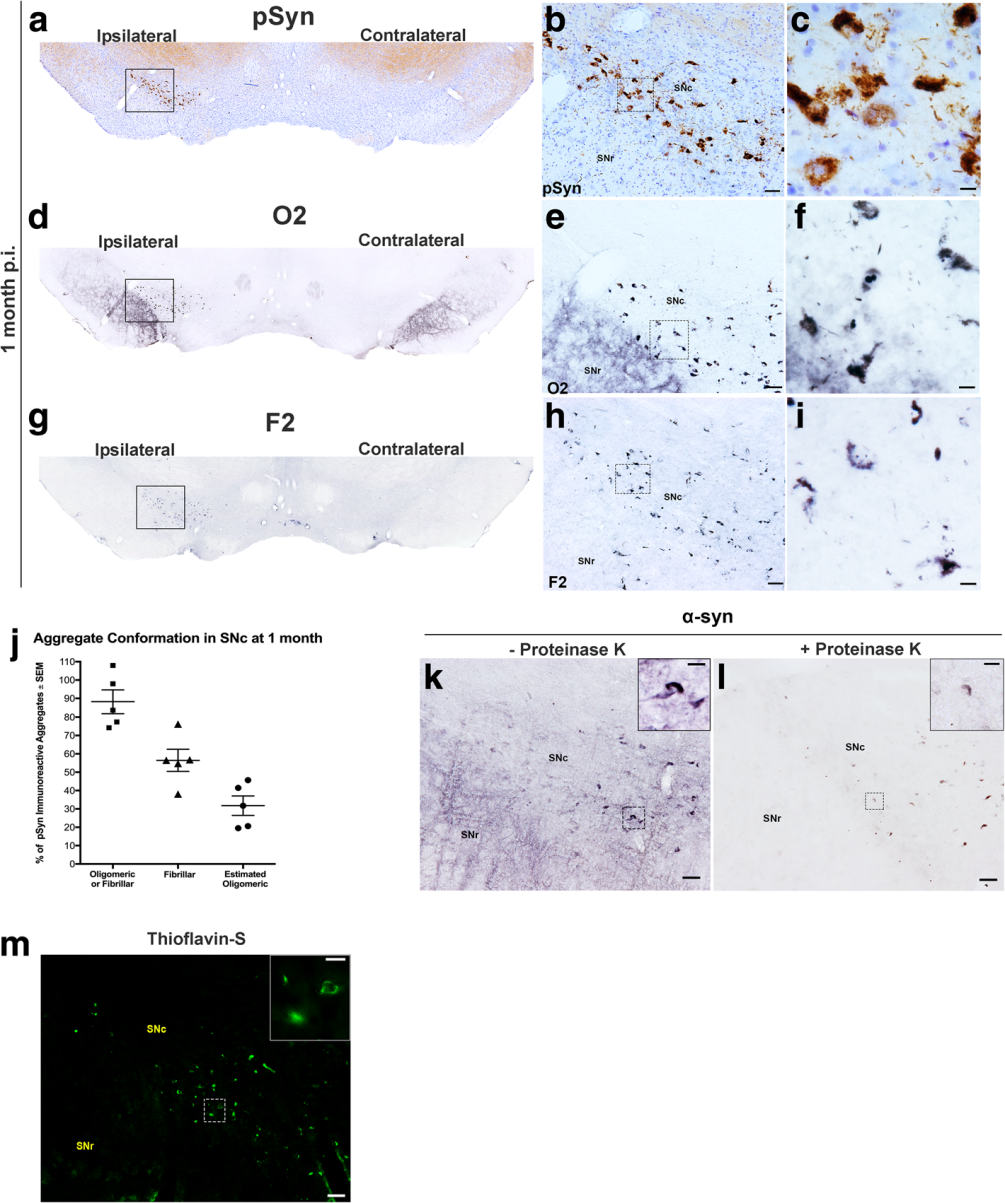
α-Syn inclusions in the SNc exhibit oligomeric and fibrillary conformations and Lewy body-like characteristics. **a**–**c** Representative images of Lewy-body-like intraneuronal pSyn inclusions in the substantia nigra pars compacta (SNc) at 1 month p.i. show pathology is localized to the ipsilateral SNc. **d**–**f** Adjacent SN tissue sections stained for O2 (oligomeric/fibrillar α-syn conformation specific) and F2 (**g**–**i**) (fibrillar-specific conformation) α-syn reveal that many intraneuronal inclusions possess mature, fibrillar inclusions of α-syn. **j** Percent of pSyn inclusions with either oligomeric/fibrillar (O2) or predominantly fibrillar (F2) immunoreactivity and estimated proportion of pSyn inclusions that are oligomeric only. Data represent mean ± SEM. Scale bars (**b**, **e**, **h**) = 50 μm, (**c**, **f**, **i**) = 10 μm. **k** Endogenous α-syn immunoreactivity in the SNc and SNr. **l** Adjacent tissue sections exposed to proteinase-K reveal the absence of soluble α-syn in the SNr and the presence of insoluble, neuronal inclusions of α-syn in the SNc. **m** Thioflavin-S fluorescence of amyloid structure present in SNc neurons. Scale bars (**k**, **l**, **m**) = 50 μm, (insets) = 25 μm. Abbreviations: α-syn = alpha-synuclein; p.i. = postinjection; pSyn = α-syn phosphorylated at serine 129; O2 = oligomeric/fibrillar α-syn antibody; F2 = fibrillar α-syn only antibody; SNc = substantia nigra pars compacta; SNr = substantia nigra pars reticulata; SEM = standard error of the mean

In our previous work [37], we reported that unilateral intrastriatal injection of mouse α-syn PFFs results in phosphorylated α-syn (pSyn) intraneuronal accumulations in several areas that innervate the striatum [45], most prominently the frontal (primary motor and somatosensory, layer 5) and insular cortices, amygdala, and SNc. Over time, accumulations increase in number in these regions. In the present study, we observed an identical pattern of pSyn accumulation in rats injected with mouse α-syn PFFs. Specifically, we observe abundant pSyn pathology bilaterally in cortical regions (layers 2/3 of the secondary motor area, insular cortex, and orbital areas; Fig. 2a). In contrast, we observed unilateral pSyn accumulation in the SNc ipsilateral to the injected striatum and complete absence of pSyn aggregates in animals injected with an equal volume of PBS or equal volume and concentration of RSA (Additional file 1: Figure S1).

Accumulation of pSyn inclusions followed a distinct temporal pattern depending on the region examined. At 2 months postinjection (p.i.), we observed abundant soma and neuritic pSyn inclusions bilaterally in the agranular insular cortex that persisted over the course of 6 months (Additional file 2: Figure S2A). Abundant pSyn accumulations were observed within the ipsilateral SNc at 2 months p.i., which remained ipsilateral and decreased in number over the course of 6 months (Additional file 2: Figure S2D–F). The abundance of pSyn inclusions in the striatum followed an opposite pattern (Additional file 2: Figure S2G–I). We observed relatively sparse pSyn inclusions in the striatum at 2 months p.i. that were primarily restricted to neurites. At 4 and 6 months p.i., the number of pSyn accumulations in striatal somata increased in abundance and also were observed in the contralateral striatal hemisphere (Additional file 2: Figure S2H–I).

### α-Syn inclusions in the SNc exhibit oligomeric, fibrillary conformations, and Lewy body-like characteristics

The oligomeric form of α-syn is proposed to be one of the toxic species [43, 46–48]. We further characterized the nature of pSyn inclusions within the SNc at 1 month p.i. using conformation-specific antibodies for oligomeric/fibrillar α-syn (O2) or fibrillar-predominant α-syn (F2) and compared that with immunoreactivity to pSyn (Fig. 2a–i [43]). When adjacent sections were quantified using unbiased stereology, we observed that 88.2 ± 6.4% of pSyn immunoreactive inclusions exhibited either an oligomeric or fibrillary conformation (O2 only). Furthermore, 56.4 ± 6.04% of nigral pSyn inclusions was detected as predominately mature, fibrillar aggregates (F2 only) with an estimated 31.7 ± 5.9% of inclusions suggested to be in an oligomeric conformation (O2 only minus F2 only; Fig. 2j). α-Syn inclusions in the SNc 2 months p.i. displayed Lewy body-like characteristics [49, 50], including resistance to proteinase-K digestion (Fig. 2i, k, l) as well as markers for ß-sheet structure as detected by thioflavin-S (Fig. 2m). Collectively, these results suggest that intrastriatal injection of mouse α-syn PFFs triggers pathological conversion of endogenous α-syn to phosphorylated, oligomeric, and fibrillary conformations in the SNc that ultimately result in insoluble, amyloid inclusions resembling Lewy bodies.

### PFF-induced synucleinopathy induces significant bilateral loss of SNc neurons

**Fig. 3:**
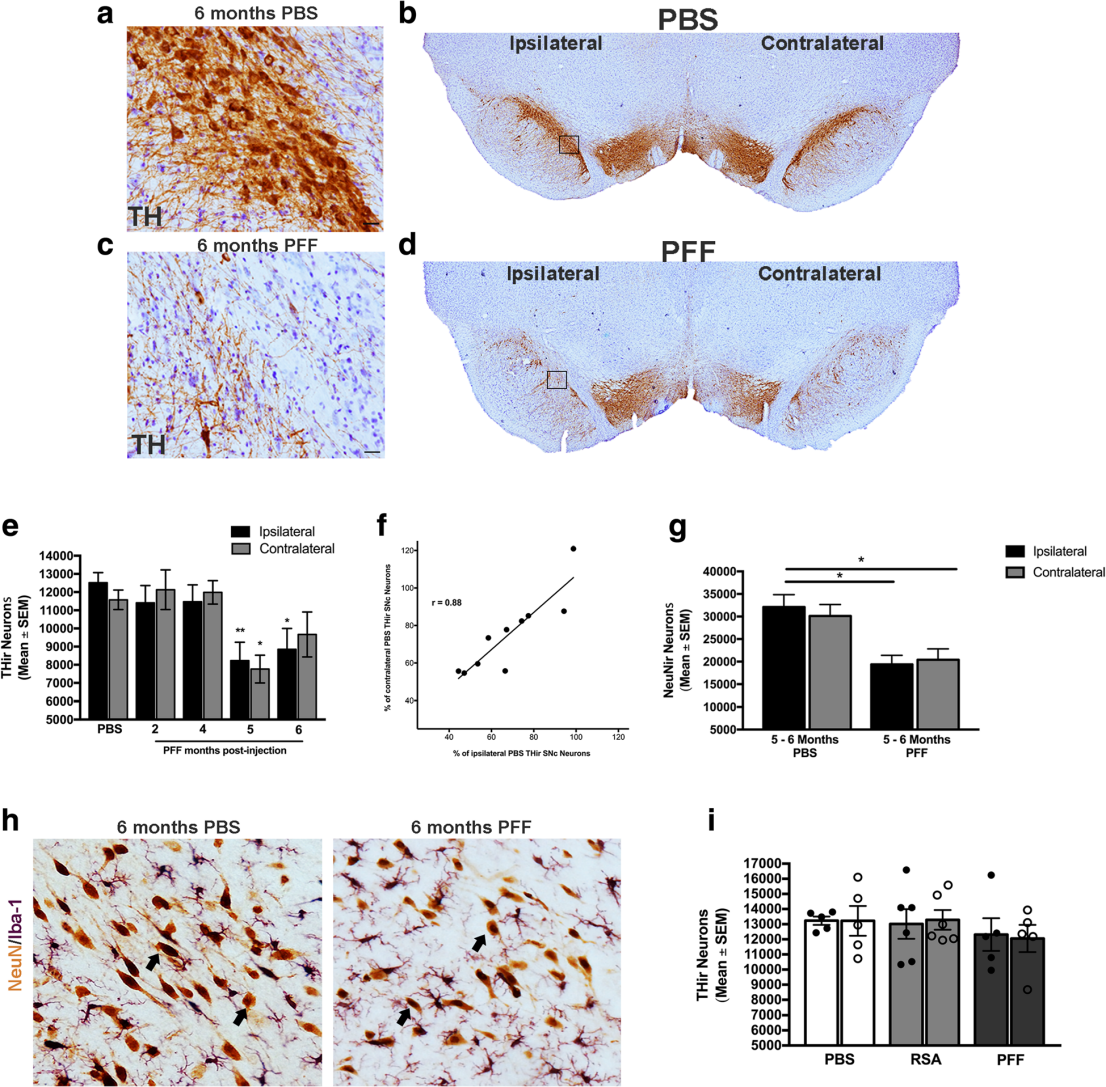
α-syn PFF-seeded synucleinopathy induces protracted, significant bilateral loss of SNc dopamine neurons. **a**–**d** Unilateral intrastriatal α-syn PFF injection induces visible loss of THir neurons (brown) in the SN at 6 months compared to an age-matched, PBS-injected control. Scale bars (**a**, **c**) = 25 μm. **e** Stereological assessment of THir neuron loss at 2, 4, 5, and 6 months following α-syn PFF or saline injection. Significant ipsilateral reduction in THir neurons was observed at 5 and 6 months postinjection compared to saline-injected controls with significant contralateral loss at 5 months *p < 0.027 compared to respective PBS hemisphere, **p < 0.013 compared to respective PBS hemisphere. **f** Correlation between extent of ipsilateral THir neuron loss and contralateral loss as compared to PBS control (*r* = 0.8855, *p* = 0.0007, *R*^2^ = 0.7842). **g** Stereological assessment of NeuNir neurons reveals overt degeneration distinct from loss of TH phenotype, *p < 0.03 compared to ipsilateral PBS. **h** Representative IHC of NeuNir neurons (brown, arrows) in the SNc in PBS (*left*) and PFF injected (*right*) animals 6 months p.i. **i** Stereological assessment of THir neurons at 2 months p.i. reveals no significant acute toxicity from injection of rat serum albumin (RSA). Data represent mean ± SEM. Abbreviations: PFF = pre-formed alpha-synuclein fibrils; p.i. = postinjection; ipsilateral = ipsilateral hemisphere relative to injection; contralateral = contralateral hemisphere relative to injection; SNc = substantia nigra pars compacta; SNr = substantia nigra pars reticulata; Neu-Nir = neuronal nuclei immunoreactive; SEM = standard error of the mean; THir = tyrosine hydroxylase immunoreactive; PFFs = pre-formed alpha-synuclein fibrils; PBS = phosphate-buffered saline; RSA = rat serum albumin

We previously observed that unilateral intrastriatal mouse α-syn PFF injections to rats resulted in bilateral nigrostriatal degeneration of THir SNc neurons within 6 months [37]. To validate this finding in our present cohort, we conducted unbiased stereology of THir SNc neurons at 2, 4, 5, and 6 months p.i. in α-syn PFF- and PBS-injected rats. Injection of PBS did not result in significant loss of THir SNc neurons at any time point (*F*_(7, 18)_ = 1.991, *p* > 0.05); thus, PBS-injected time points were combined for comparison to PFF-injected rats between identical hemispheres (ipsilateral PBS = 12,518 ± 554; contralateral PBS 11,577 ± 536). Similar to our previous studies, we observed significant, bilateral reduction (~ 35%) in SNc THir neurons (Fig. 3a–e). Specifically, the number of SNc THir neurons ipsilateral to α-syn PFF injection at both 5 (8227 ± 1015) and 6 months (8851 ± 1148) p.i. was significantly reduced compared to PBS control rats (*F*_(5, 36)_ = 4.297, *p* < 0.027, Fig. 5e). Within the contralateral SNc, significantly fewer THir neurons were observed 5 months following α-syn PFF injection (*F*_(5, 36)_ = 5.782, *p* < 0.013) with a non-significant reduction in the contralateral SNc observed at 6 months (*p* > 0.05). A positive correlation existed between the extent of ipsilateral THir SNc neuron loss and the extent of contralateral loss of THir SNc neurons (*r* = 0.8855, *p* = 0.0007, *R*^2^ = 0.7842, Fig. 3f).

Lastly, to confirm whether reductions in THir neurons induced by PFF injection represented phenotype loss or overt degeneration, unbiased stereology of NeuN-ir neurons in the SNc was conducted in PFF- or PBS-treated groups at 5 and 6 months p.i. No significant differences were observed within the corresponding hemisphere between 5 and 6 months due to either PBS or PFF injection (PBS: *F*_(3, 4)_ = 1.238, *p* > 0.05; PFF: *F*_(3, 8)_ = 0.3986, *p* > 0.05). Therefore, the 5- and 6-month time points were combined into one time point. The number of SNc NeuN-ir neurons ipsilateral to PFF injection was significantly reduced compared to either the ipsilateral or contralateral hemisphere of PBS-injected rats (*F*_(3, 16)_ = 7.089, *p* < 0.02). The number of NeuN-ir neurons in the contralateral SNpc of PFF injected rats was significantly reduced compared to the ipsilateral SNc of PBS injected rats (*p* < 0.0319). When compared to the contralateral SNc of PBS-injected rats, NeuN-ir neurons were reduced yet did not reach significance (*p* = 0.0563, Fig. 3g–i). Overall, our results replicate our previous findings that intrastriatal α-syn PFF injection results in significant bilateral reductions in THir and NeuN-ir SNc neurons over the course of 6 months [37].

In a control experiment, we examined whether intrastriatal injection of an exogenous protein taken up by neurons [51, 52], rat serum albumin (RSA), induced an inflammatory response in the absence of intracellular pSyn accumulation. To rule out acute toxicity induced by RSA, THir SNc neurons were quantified at 2 months after injection. No significant differences in THir SNc neurons were observed due to PBS, RSA, or PFF injection in either the ipsilateral or contralateral hemisphere (*F*_(5, 26)_ = 0.3731, *p* > 0.05, Fig. 3j). These results demonstrate that RSA injection, used as an additional control treatment, did not compromise the survival of THir SNc neurons.

### Phosphorylated α-syn inclusions peak in the SNc at 2 months and significantly decrease in number during the 5–6-month interval of SNc degeneration

**Fig. 4:**
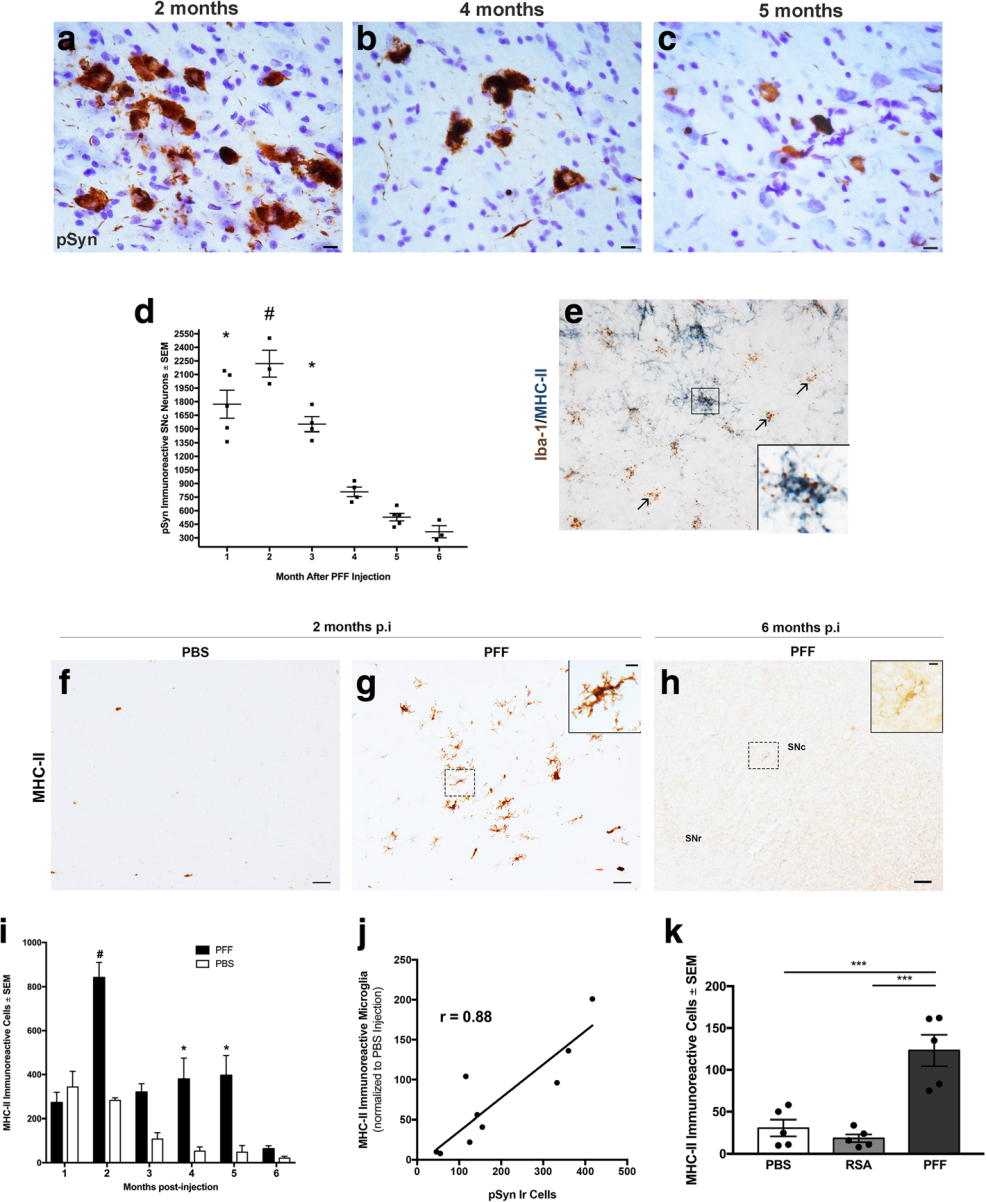
Antigen-presenting MHC-II immunoreactive (MHC-IIir) microglia increase in the SNc in association with peak accumulation of α-syn inclusions but are limited during the interval of degeneration. **a**–**c** Representative images of pSyn inclusions in the SNc at 2, 4, and 5 months p.i.; scale bar (**a**–**c**) = 10 μm. **d** Stereological assessment of pS129 containing neurons in the substantia nigra in PFF animals at 1, 2, 3, 4, 5, and 6 months p.i.; *p ≤ 0.001 compared to 4, 5, and 6 months. ^#^*p* ≤ 0.006 compared to 3, 4, 5, and 6 months. pSyn inclusions decrease over time in association with neuronal loss. **e** Major-histocompatibility complex-II (MHC-II; blue) protein colocalizes with ionized calcium-binding adaptor molecule 1 mRNA (brown) within microglia. **f**, **g** Representative images of MHC-II antigen-presenting microglia in the SN at 2 months in PBS- and PFF-injected rats and 6 months post-PFF injection (**h**); scale bar (**f**–**h**) = 50 μm, insets = 10 μm. **i** Stereological assessment of MHC-IIir microglia in the SN reveals MHC-IIir microglia are significantly higher in PFF vs. PBS animals at 2, 4, and 5 months *p < 0.006. More MHC-IIir microglia are evident in 2-month PFF animals vs. all other PFF time points ^#^*p* < 0.02. Notably, MHC-IIir microglia peak at the same time pSyn aggregation peaks (**d**, **i**). **j** Number of MHC-II immunoreactive microglia correlated with number of SNc neurons with intraneuronal pSyn inclusions (*r* = 0.8858, *p* = 0.0015, *R*^2^ = 0.7846). **k** In a follow-up study, intrastriatal injection of rat serum albumin (RSA) does not impact numbers of MHC-IIir microglia compared to PBS *p* > 0.05. Injection of PFFs in this second cohort confirmed previous observations of a significant increase in MHC-IIir microglia compared to PBS or RSA at 2 months p.i. *** *p* ≤ 0.0006. Abbreviations: p.i. = postinjection; PFFs = pre-formed alpha-synuclein fibrils; PBS = phosphate-buffered saline; RSA = rat serum albumin; MHC-II = major-histocompatibility complex-II; Iba-1 = ionized calcium binding adaptor molecule 1; ir = immunoreactive; SNc = substantia nigra pars compacta; SEM = standard error of the mean

We determined the time course (1–6 months p.i.) of phosphorylated α-syn (pSyn) accumulation in the SNc following intrastriatal α-syn PFF injection at monthly intervals. pSyn inclusions were observed in the SNc ipsilateral to injection in all α-syn PFF-injected rats, with the number of inclusions varying based on time point after injection (Fig. 4a–d). Inclusions were most abundant at months 1, 2, and 3, with all three time points exhibiting significantly higher α-syn inclusions compared to the interval of SNc degeneration at months 4, 5, and 6 (Figs. 3e, g and 4d; *F*_(5, 18)_ = 2.251, *p* ≤ 0.001). The number of intraneuronal α-syn inclusions in the SNc was significantly greater at 2 months p.i. compared to all other time points except the 1-month time point (Fig. 4d; *p* ≤ 0.006). At 2 months, approximately 2220 ± 148.6 SNc neurons possessed pSyn inclusions. By comparison, a loss of ≈ 3804 THir SNc neurons ipsilateral to PFF injection was observed at 5–6 months. These results suggest that pSyn inclusion formation in the SNc between 1 and 3 months after PFF injection precedes degeneration of the SNc neurons at 5–6 months p.i.

### MHC-II immunoreactive (MHC-IIir) microglia increase in the SNc in association with accumulation of α-syn inclusion but are decreased during the interval of degeneration

MHC-II expression on microglia is associated with co-expression of pro-inflammatory genes such as TNF, IL-1β, and CD80 as well as proinflammatory cytokine secretion [13–15]. We quantified MHC-II immunoreactive (MHC-IIir) microglia within an adjacent series of SNc tissue sections at months 1, 2, 3, 4, 5, and 6 after unilateral α-syn PFF or PBS intrastriatal injection in order to examine neuroinflammation. Double labeling for MHC-II protein and Iba-1 mRNA confirmed the identity of MHC-IIir cells to be microglia (Fig. 4e).

MHC-IIir microglia were observed in the SNc ipsilateral to injection in both α-syn PFF and PBS control rats at all time points. No MHC-IIir microglia were observed in the contralateral SNc. However, the magnitude of MHC-IIir microglia varied over time and followed a nearly identical pattern to that observed with pSyn inclusion accumulation. At the one-month time point, no significant differences were observed between the number of MHC-IIir microglia in the ipsilateral SNc of PBS controls compared to α-syn PFF-injected rats (*F*_(11, 45)_ = 17.45, *p* > 0.05), presumably reflecting a non-specific response to injection (Fig. 4i). However, significantly higher numbers of MHC-IIir microglia were observed in the ipsilateral SN of α-syn PFF-injected rats compared to PBS-injected control rats at months 2, 4, and 5 (*p* < 0.006, Fig. 4f, g, i). The peak of MHC-IIir microglia occurred in the SN 2 months following α-syn PFF injection (*p* < 0.02 compared to PFF-injected rats all other time points), corresponding to the time point when the greatest number of SNc neurons possesses α-syn aggregates (Fig. 4d, g, i). In contrast, significantly fewer MHC-IIir microglia were observed in PFF-injected rats at months 5 and 6, corresponding to the interval of SNc THir neuron loss, although these numbers were still significantly higher than PBS-injected controls (*p* < 0.006, Figs. 3e and 4i). There was a positive correlation between the number of MHC-IIir microglia and the number of SNc neurons possessing pS129 α-syn inclusions in the SN (*r* = 0.8858, *p* = 0.0015, *R*^2^ = 0.7846, Fig. 4j, months 2, 4, and 6).

To confirm these findings, we repeated injections in a separate cohort of animals with rat serum albumin (RSA) as an additional control group for neuronal uptake of exogenous protein in the absence of pSyn accumulation, as PBS injection only controls for needle insertion into the parenchyma. As in previous cohorts, α-syn PFF injection resulted in a significant increase in MHC-IIir microglia in the SN at 2 months p.i. (Fig. 4k, *p* ≤ 0.0006). Injection of RSA resulted in similar numbers of MHC-IIir microglia as observed in PBS-injected control rats. No acute neurotoxicity was observed in RSA-injected animals at 2 months p.i. (Fig. 3i). Collectively, these results reveal that the preponderance of MHC-II expression in SN microglia is associated with pSyn α-syn inclusions at early time points, however is significantly attenuated during the interval of THir SNc degeneration.

### pSyn inclusions in the SNc are associated with a reactive microglial morphology in the adjacent SNr

**Fig. 5:**
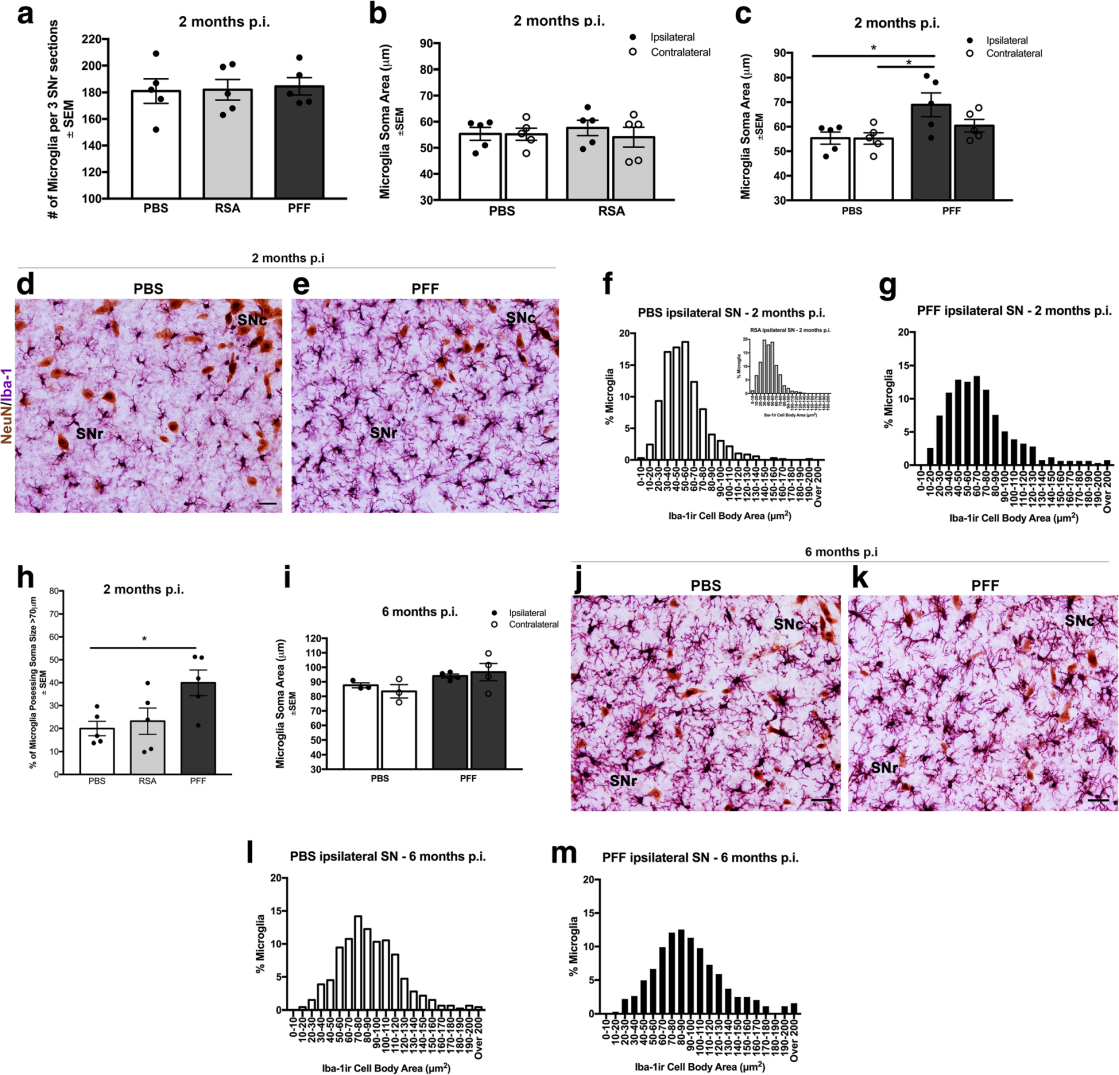
SNr microglia exhibiting reactive morphology are associated with pSyn inclusion-bearing neurons in the SNc. **a** Total number of microglia did not differ between PBS-, RSA-, and PFF-injected animals, *p* > 0.05. **b** Microglia soma area in the ipsilateral and contralateral SNr did not differ significantly between animals receiving intrastriatal injections of PBS or RSA, *p* > 0.05. **c** Microglia soma area was significantly increased at 2 months in the ipsilateral SNr, when peak numbers of pSyn aggregates are present in nearby SNc neurons as compared to PBS-injected animals, *p = 0.02. **d**, **e** Representative images of SN sections dual labeled for Iba-1 immunoreactive microglia (purple) and NeuN-ir neurons (brown) 2 months following either intrastriatal PBS or α-syn PFF-injected rats. α-Syn PFF-injected rats exhibit larger cell bodies and increased number and thickness of processes. **f**, inset Distribution of microglia soma area measurements 2 months following intrastriatal PBS or RSA injection illustrated as a percent of total microglia quantified. **g** Distribution of microglia soma area measurements 2 months following α-syn PFF injection illustrated as a percent of total microglia quantified. **h** Percent of total microglia quantified for soma area analysis with cell body areas > 70 μm at 2 months. Microglia in PFF-injected rats possessed significantly more microglia with cell bodies larger than > 70 μm compared to PBS-injected rats, *p = 0.03. **i** At 6 months p.i. microglia soma area in the ipsilateral and contralateral SNr did not differ significantly between rats receiving either PBS or α-syn PFF intrastriatal injections, during the interval of ongoing degeneration in the SNc of PFF-injected animals, *p* > 0.05. **j**, **k** Representative images of SN sections dual labeled for Iba-1 immunoreactive microglia (purple) and NeuN-ir neurons (brown) at 6 months p.i. exhibit a hyper ramified morphology, regardless of treatment. **l**, **m** Distribution of microglia soma area measurements 6 months p.i in PBS- and PFF-injected rats as a percent of total microglia quantified. Scale bars (**d**, **e**, **j**, **k**) = 25 μm. Data represent mean ± SEM. Abbreviations: p.i. = postinjection; PFFs = pre-formed alpha-synuclein fibrils; PBS = phosphate-buffered saline; RSA = rat serum albumin; Iba-1 = ionized calcium-binding adaptor molecule 1; ir = immunoreactive; NeuN-ir = neuronal nuclei immunoreactive; ipsilateral = ipsilateral hemisphere relative to injection; contralateral = contralateral hemisphere relative to injection; SNc = substantia nigra pars compacta; SNr = substantia nigra pars reticulata; SEM = standard error of the mean

**Fig. 6:**
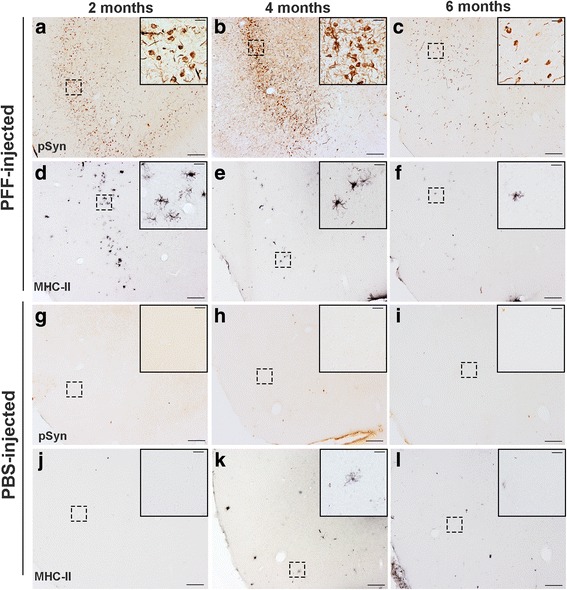
Microglia expressing MHC-II are associated with pSyn inclusions in the agranular insular cortex. **a**–**c** Representative images of pSyn accumulation in the agranular insular cortex at 2, 4, and 6 months p.i. Pathology is primarily localized to the soma at 2 months (**a**), with increased neuritic pathology by 4 months (**b**) and an observable decrease overall by 6 months, possibly due to death of neurons. **c** No pSyn inclusions were observed in PBS-injected animals (**g**–**i**). **d**–**f** A similar pattern was observed in MHC-II expression on microglia. Peak numbers of MHC-IIir microglia were observed at 2 months p.i. (**d**), decreased at 4 months (**e**), and virtually absent by 6 months (**f**). **j**–**l** Few to no MHC-IIir microglia were observed in PBS injected animals at any time point, suggesting that MHC-II expression occurs in response to accumulation of pSyn inside neurons. Abbreviations: p.i. = postinjection; pSyn = α-syn phosphorylated at serine 129; PFFs = pre-formed alpha-synuclein fibrils; PBS = phosphate-buffered saline; MHC-II = major-histocompatibility complex-II; ir = immunoreactive

The number and distribution of MHC-IIir microglia in the SN suggested that not all microglia were expressing MHC-II. We next used Iba-1 immunoreactivity to examine the entire microglia population within an adjacent series of SN tissue sections at 2 and 6 months after α-syn PFF, RSA, or PBS intrastriatal injection (Fig. 5). Quantitation of the number of Iba-1 immunoreactive (Iba-1ir) microglia in the adjacent SNr revealed no significant differences in microglial number due to α-syn PFF, RSA, or PBS injection at either 2 months (Fig. 5a) or 6 months p.i. (2 months: *F*_(3, 16)_ = 0.2637, *p* > 0.05; 6 months: *F*_(3, 10)_ = 0.2427, *p* > 0.05). No significant differences were observed in microglial soma area in the SNr due to intrastriatal RSA injections at the 2-month time point (*F*_(3, 16)_ = 0.256, *p* = 0.855, Fig. 5b). At the 2-month time point coinciding with the peak of pSyn α-syn inclusion accumulation in the SNc, we observed an appreciable increase in the soma size and thickness and number of microglial processes in the SNr of PFF-injected rats compared to a more classically quiescent microglial morphology observed in control injected rats. Specifically, in the ipsilateral SNr of rats 2 months following α-syn PFF injection, the average microglia cell body area was significantly larger compared to PBS-injected rats (*F*_(3, 16)_ = 4.016, *p* = 0.02, Fig. 5c–e). Microglia soma area varied in all conditions between ≈ 10–200 μm^2^, with a significantly greater percentage of microglia > 70 μm^2^ observed in the SNr of rats possessing SNc pSyn α-syn inclusions at 2 months compared to rats injected with PBS (Fig. 5f–h, *F*_(2, 12)_ = 4.613, *p* = 0.03).

At 6 months p.i., a time point corresponding to the time point of very few pSyn α-syn inclusions and immediately following loss of SNc neurons, no significant differences in microglia soma size were observed between α-syn PFF- and PBS-injected rats (*F*_(3, 10)_ = 2.089, *p* > 0.05, Fig. 5i–m). Of note, the average microglial soma area in PBS-injected rats at 6 months (rats 8 months of age) was significantly larger than PBS-injected rats at 2 months (4 months of age) suggesting an age-related increase (*F*_(3, 12)_ = 37.00, *p* < 0.001). The distribution of microglia soma areas between PFF and PBS rats 6 months following injection also appeared similar (Fig. 5l, m) with an apparent age-related effect [53–55] reflected in a greater percentage of microglia > 70 μm^2^ in 8-month-old rats compared to 4-month-old rats.

Overall, our finding that the peak time point of SNc pSyn α-syn inclusions is associated with a significant increase in microglia soma size suggests that synucleinopathy in the SNc triggers early disturbances in local microglia. The interval in which we observe this synucleinopathy-induced reactive microglial morphology is 3 months prior to loss of SNc neurons (Fig. 3e, g) suggesting that reactive microglia have the potential to contribute to vulnerability of SNc neurons to degeneration.

We also examined a series of sections throughout the SN and striatum at 2, 4, and 6 months p.i. in α-syn PFF- and PBS-injected rats for the presence of cluster of differentiation 68 (CD68) which labels both phagocytic microglia and infiltrating macrophages. While a few CD68-ir cells were observed in blood vessels, no CD68-ir cells were observed in the parenchyma during any of the time points examined (data not shown). The lack of CD68 immunoreactivity in the parenchyma of the SN or striatum at any time point suggests that the magnitude of synucleinopathy and subsequent degeneration produced in the α-syn PFF model does not trigger microglial phagocytic activity.

### MHC-IIir microglia in the agranular insular cortex are associated with the accumulation of α-syn inclusions

We also examined the time course of pSyn inclusion accumulation and MHC-II expression on microglia in the agranular insular cortex, as this region possesses abundant Lewy-body like pathology in our model and is implicated in non-motor symptoms in PD [56]. At 2 months p.i., we observe abundant pSyn inclusions primarily localized to the somata (Fig. 6a), with increased neuritic pathology evident by 4 months (Fig. 6b). Interestingly, an observable decrease in both neuritic and somata inclusions is evident by 6 months (Fig. 6c). We observe a similar temporal pattern of MHC-IIir microglia as described for the SN: the highest number of MHC-IIir microglia is observed at 2 months p.i., when pSyn inclusions first peak, and a decrease in MHC-IIir microglia in association with reductions in the number of pSyn inclusions. Interestingly, a decrease in MHC-IIir microglia is observed between 2 and 4 months p.i. when pSyn pathology becomes more abundant with the appearance of neuritic inclusions. These observations suggest that MHC-II is upregulated as a *first response* to *formation* of pSyn inclusions and is not sustained over time, despite a secondary increase in synuclein burden. Few to no MHC-IIir microglia were observed in PBS-injected animals (Fig. 6g–l), strengthening the concept that MHC-IIir on microglia is induced by initial accumulation of pSyn.

### MHC-IIir microglia in the striatum are not associated with the accumulation of α-syn inclusions

Lastly, we examined the time course of accumulation of pSyn inclusions and number of MHC-IIir microglia in the striatum in rats that received unilateral α-syn PFF or PBS intrastriatal injection. As reported previously [37], the pattern of pSyn α-syn inclusion accumulation in the striatum is strikingly different from accumulation in the SNc. At 2 months, α-syn inclusions in cell bodies in the ipsilateral striatum are relatively sparse and pSyn α-syn immunoreactivity appeared primarily localized to neurites, presumably in terminals from the SNc (Additional file 1: Figure S1A). Over the course of the 4 months, pSyn α-syn inclusions in cell bodies in the striatum increase in number, involving the contralateral hemisphere as well, with the greatest number of pSyn α-syn inclusions observed in the ipsilateral striatum at the 6-month time point (Additional file 1: Figure S1C). In adjacent striatal tissue sections, we examined the temporal pattern of MHC-IIir microglia following unilateral α-syn PFF or PBS intrastriatal injection. Early after injection, at 2 weeks, 1 month, and 2 months, abundant MHC-IIir microglia were observed in the ipsilateral striatum in proximity to the injection sites in both PFF- and PBS-injected rats (Additional file 1: Figure S1D, G). No differences were observed between treatment groups at the 1-month time point, but the magnitude of the MHC-II response appeared slightly larger in PFF-injected rats compared to the PBS-injected rats at the 2-month time point. However, at 4 and 6 months, during the interval of continuing accumulation of pSyn α-syn inclusions in the striatum, the number of MHC-IIir microglia decreased dramatically with no differences observed in the small number of MHC-IIir microglia observed in both treatment groups (Additional file 1: Figure S1E, F, H, I). These results suggest that the acute microglial response to the PFF injectate may differ from the acute response to the surgical injection alone but that the subsequent increase α-syn inclusion load within neurons in the striatum does not trigger a second wave of microglial MHC-II immunoreactivity.

## Discussion

**Fig. 7:**
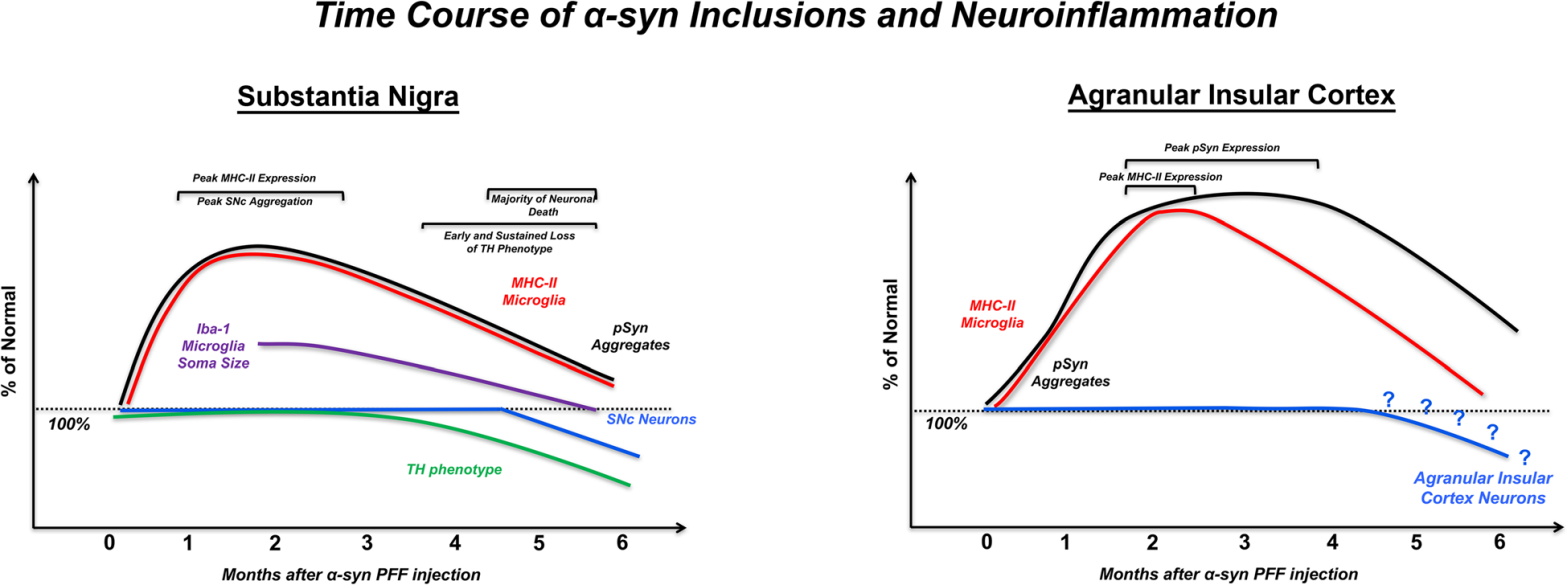
Regional timelines of synucleinopathy, neuroinflammation, and degeneration in the substantia nigra and agranular insular cortex following intrastriatal α-syn PFF injection. (Left): Early accumulation of phosphorylated inclusions of α-syn (peak at 2 months) in the substantia nigra leads to loss of TH phenotype and eventual loss of nigrostriatal dopamine neurons by 5–6 months p.i. In the SN, the pattern of microgliosis similarly follows that of pSyn: microglia in the adjacent SNr exhibit a reactive morphology at 2 months p.i. when nearby SNc neurons possess the greatest number of SNc pSyn inclusions. Interestingly, MHC-IIir antigen-presenting microglia in the SNc also peak at 2 months p.i., again coinciding with the greatest number of pSyn intraneuronal inclusions and decrease over time to near non-detectable levels during the interval of degeneration, suggesting a relationship between pathological α-syn and inflammation. (Right): Early accumulation of pSyn inclusions occurs between 2 and 4 months, with inclusions primarily localized to the somata at 2 months, an increase in neuritic inclusions at 4 months, and an observable decrease of overall pSyn pathology at 6 months, suggesting that similar to the SN, neurons in the agranular insular cortex harboring inclusions eventually die off. MHC-II immunoreactivity follows a similar pattern to that observed in the SN, with peak expression observed at 2 months p.i., and decreased over the course of 6 months

In the present study, intrastriatal injection of α-syn PFFs to rats resulted in widespread accumulation of phosphorylated α-syn inclusions in several areas that innervate the striatum, as previously reported in rats and mice [37, 57]. Further examination of the inclusions formed in the SNc revealed that they share many key features with Lewy bodies and were most abundant between months 1–3 after intrastriatal α-syn PFF injection, peaking at 2 months. The magnitude of ipsilateral SNc neurons bearing α-syn inclusions 1–3 months after α-syn PFF injection approximated the magnitude of loss of ipsilateral SNc neurons observed at 5–6 months, suggesting a direct relationship between α-syn inclusion accumulation and degeneration of SNc neurons. Synucleinopathy-specific MHC-II expression in the ipsilateral SNc similarly peaked in the SN at 2 months and was associated with a reactive microglial morphology, characterized by significantly larger soma size, 3 months prior to degeneration. Surprisingly, although the period of nigral degeneration was associated with an increased MHC-II signal relative to controls, MHC-II immunoreactivity during the period of degeneration was significantly decreased relative to MHC-II immunoreactivity during the earlier peak of synucleinopathy. Overall, the temporal pattern of peak Lewy body-like inclusion formation was associated with peak neuroinflammation in the SN, both of which appear months prior to loss of SNc neurons. These results suggest that an increase in MHC-II may be a first-response mechanism to initial accumulation of intracellular α-syn and that reactive microglia have the potential to contribute to vulnerability of SNc neurons to degeneration (Fig. 7, left).

Within the agranular insular cortex, we observe pSyn primarily localized to the somata at 2 months, followed by an increase in neuritic pathology by 4 months, and a significant decrease overall in pSyn immunoreactivity at 6 months (Fig. 7, right). This suggests that similar to the SN, neurons in the agranular insular cortex harboring inclusions die over the course of 6 months. Moreover, a similar pattern of MHC-II expression on microglia is observed: numbers of MHC-IIir microglia peak when pSyn pathology is most abundant and decrease over time. Although this has not been systematically quantified in the present study, future studies investigating inflammation and degeneration in this area following intrastriatal α-syn PFF injection are warranted, as the insula has been implicated in the manifestations of non-motor symptoms in human PD [56, 58, 59].

Within the striatum, the site of α-syn PFF injection, we observed a distinctly different pattern of accumulation of α-syn inclusions compared to the SNc (Additional file 3: Figure S3J). We observed a dissociation between α-syn inclusion load and MHC-II immunoreactivity in the striatum (Additional file 3: Figure S3). The procedure of intrastriatal injection itself triggered an acute increase in MHC-II immunoreactive microglia that appeared to be slightly enhanced by the presence of the PFF injectate; however, the presence of MHC-II decreased dramatically over time in all conditions despite an ever increasing α-syn inclusion load. Compared to the SNc, the accumulation of α-syn inclusions in the striatum is delayed with no loss of striatal neurons observed at 6 months [37] and it is unknown whether degeneration of striatal neurons ultimately occurs at later time points. It is unclear whether α-syn inclusion load increases and peaks past the 6-month time point and whether the presence of MHC-II immunoreactive microglia may have similarly tracked with a future peak. The results in the striatum illustrate the necessity of determining the role of an acute inflammatory response to local injection and that the magnitude of this response may prevent the reactive microgliosis associated with pathological α-syn inclusions. Further, these striatal results suggest that α-syn inclusions do not automatically trigger reactive microgliosis and that other factors including rate of inclusion formation, impending cytotoxicity, local environment, or microglia and astrocyte density [42] may be involved in determining the neuroinflammatory cascade of events.

In response to persistent neuronal stress and protein accumulation such as α-syn aggregation, microglia can become chronically activated, proliferate, migrate, secrete pro-inflammatory cytokines and reactive oxygen species, and ultimately contribute to neuronal injury in an uncontrolled, feed-forward manner [20, 60–63]. Microglia can also phagocytose both living and dead neurons [64–66]. The observation that α-syn inclusion triggered MHC-II expression on microglia in the SN prior to degeneration indicates that neuroinflammation may contribute to the mechanism of pathophysiology. However, it is unlikely that microglial activation is the sole arbiter of degeneration given that neuronal death can result from α-syn PFF-induced intraneuronal inclusions in cultures in the absence of microglia [40]. A more likely scenario involves neuroinflammation contributing to or accelerating nigrostriatal degeneration with properties unique to the nigral environment [67–72] adding to the cascade of events. Future studies investigating the secretion of proinflammatory and anti-inflammatory cytokines in the SN at time points prior to and following nigral degeneration is warranted.

Human PD studies examining neuroinflammation suggest involvement of both the local brain immune response and the adaptive immune system in PD [73–75]. In the present study, we did not examine the possibility of peripheral immune cell infiltration beyond phagocytic CD68+ macrophages, which were not detected in the parenchyma in PFF-injected animals at any stage, and data from human tissue regarding the presence of CD68+ macrophages in human PD is limited [9]. However, microglia are considered to be the principle antigen-presenting cell within the brain and MHC-II expression is associated with the recognition of CD4+ T-helper cells. It is possible that CD4+ T cells participate in the response to α-syn inclusions in the SN; however, whether the net effect of CD4+ T cells is neurodegenerative or neuroprotective requires further systematic evaluation [76]. In addition, MHC-I expression by SNc neurons was observed in association with α-syn overexpression [77] and may similarly be expressed by α-syn inclusion-bearing SNc neurons. In addition, it was recently shown that T lymphocytes isolated from PD patients recognize specific α-syn peptides [78], strengthening the concept that neuroinflammation can be induced by α-syn and potentially involved early in PD progression.

While the concept that MHC-II is involved in PD and correlates with α-syn burden [9] has existed for several decades, our results are the first to systematically evaluate the time course of *endogenous* pSyn accumulation and microglial MHC-II expression prior to and after nigrostriatal degeneration has occurred. Our results indicate that MHC-II expression in the SNc is increased at early time points in which SNc neurons possess pSyn inclusions, and is relatively sparse during the interval of nigral degeneration, suggesting that MHC-II is a response to initial inclusion formation. Future studies investigating the direct impact of increased or decreased MHC-II expression on the magnitude of degeneration and what role, if any, peripheral T cells play in disease progression are warranted.

Our results and the well-characterized rat α-syn PFF model will facilitate future studies to provide key mechanistic insights into the specific relationship between pathological α-syn inclusions, neuroinflammation, and degeneration in sporadic PD.

## Conclusions

Accumulation of intraneuronal inclusions of phosphorylated α-syn induces increased MHC-II expression and reactive microgliosis in the substantia nigra months prior to dopaminergic cell death, suggesting that microglia may be a contributor to rather than only a consequence of nigral degeneration. These results will facilitate future studies to provide key mechanistic insights into the specific relationship between pathological α-syn inclusions, neuroinflammation, and degeneration in sporadic PD.

## Additional files

Additional file 1: **Figure S1.** Unilateral intrastriatal injection of α-syn PFFs, but not RSA or PBS, induces bilateral cortical and unilateral SNc Lewy-body like inclusions of phosphorylated α-syn (pSyn). (a) pSyn pathology is observed bilaterally in cortical areas after unilateral injection of α-syn PFFs, namely in layers 2/3 and orbital and agranular insular cortices. (b) Injection of PBS or (c) RSA did not induce pSyn accumulation. (d) pSyn accumulation in the ipsilateral substantia nigra pars compacta (SNc) at 2 months postinjection, with no evidence of pSyn inclusions in the contralateral SNc. Scale bars (A–D) = 50 μm. Abbreviations: α-syn = alpha-synuclein; PFFs = pre-formed alpha-synuclein fibrils; PBS = phosphate-buffered saline; RSA = rat serum albumin; pSyn = α-syn phosphorylated at serine 129. (TIF 117729 kb)

Additional file 2: **Figure S2.** Unilateral intrastriatal injection of α-syn PFFs induces widespread accumulation of Lewy-body like inclusions of phosphorylated α-syn (pSyn). Representative images illustrating the time course of pSyn pathology in regions innervating the striatum. (a–c) pSyn pathology in the ipsilateral agranular insular cortex localized to both the soma and neurites at 2 months p.i. (postinjection) that over time becomes primarily localized to the soma; scale bar = 50 μm, inset = 10 μm. (d–f) Ipsilateral accumulation of pSyn in the substantia nigra peaks at 2 months and becomes less abundant over time as neurons degenerate; scale bar = 200 μm, inset = 25 μm. (g–i) In contrast to other areas, pSyn in the striatum is primarily localized to neurites at 2 months and becomes more abundant and localized to the soma over time, scale bar = 50 μm, inset = 10 μm. Abbreviations: α-syn = alpha-synuclein; PFFs = pre-formed alpha-synuclein fibrils; pSyn = α-syn phosphorylated at serine 129; p.i. = postinjection. (TIF 33472 kb)

Additional file 3: **Figure S3.** Antigen-presenting MHC-IIir microglia are not associated with peak of intraneuronal inclusions of pSyn in the striatum. Progression of pSyn pathology and MHC-IIir microglia in the striatum. (a) At 2 months p.i., pSyn inclusions are localized to neurites, presumably representing terminals from the SNc. (b–c) Over time pSyn inclusions become primarily localized to the soma of striatal neurons. (d) Abundant MHC-IIir microglia in the striatum primarily localized around the α-syn PFF injection site at 2 months. (e–f) MHC-IIir microglia in the striatum are largely absent during continuing accumulation of intraneuronal pSyn inclusions at 4 months (e) and 6 months (f) p.i. (g) Intrastriatal injection of PBS results abundant MHC-IIir microglia in the striatum localized near the site of injection at 2 months p.i., although appearing less abundant than MHC-IIir microglia in the striatum of α-syn PFF rats at the same time point (d). (h) MHC-IIir microglia are similarly absent from the parenchyma by 4 months (h) and 6 months p.i (i). Scale bars A–I = 50 μm. Abbreviations: p.i. = postinjection; PFFs = pre-formed alpha-synuclein fibrils; PBS = phosphate-buffered saline; pSyn = α-syn phosphorylated at serine 129, MHC-IIir = major-histocompatibility complex-II immunoreactive. (TIF 112368 kb)

## Abbreviations

6-OHDA: 6-Hydroydopamine
AAV: Adenoassociated virus
CD68: Cluster of differentiation molecule 68
DMS: Dorsomedial striatum
Iba-1: Ionized calcium-binding adaptor molecule-1
IHC: Immunohistochemistry
MHC-II: Major histocompatibility complex II
MPTP: 1-Methyl-4-phenyl-1,2,3,6-tetrahydropyridine
NeuN: Neuronal nuclei
PBS: Phosphate-buffered saline
PD: Parkinson’s disease
PFFs: Alpha-synuclein pre-formed fibrils
pSyn: Alpha-synuclein phosphorylated at serine 129
RSA: Rat serum albumin
SNc: Substantia nigra pars compacta
SNr: Substantia nigra pars reticulata
TH: Tyrosine hydroxylase
VLS: Ventrolateral striatum
α-syn: Alpha-synuclein
