# Correction to: Lewy body-like alpha-synuclein inclusions trigger reactive microgliosis prior to nigral degeneration

**DOI:** 10.1186/s12974-018-1202-9

**Published:** 2018-05-29

**Authors:** Megan F. Duffy, Timothy J. Collier, Joseph R. Patterson, Christopher J. Kemp, Kelvin C. Luk, Malú G. Tansey, Katrina L. Paumier, Nicholas M. Kanaan, D. Luke Fischer, Nicole K. Polinski, Olivia L. Barth, Jacob W. Howe, Nishant N. Vaikath, Nour K. Majbour, Omar M. A. El-Agnaf, Caryl E. Sortwell

**Affiliations:** 1 Department of Translational Science and Molecular Medicine, Michigan State University, 400 Monroe Avenue NW, Grand Rapids, MI 49503-2532 USA; 2 Neuroscience Graduate Training Program, Michigan State University, Grand Rapids, MI USA; 3 MD/PhD Program, Michigan State University, Grand Rapids, MI USA; 4 Mercy Health Hauenstein Neuroscience Medical Center, Grand Rapids, MI USA; 5 Center for Neurodegenerative Disease Research, Department of Pathology and Laboratory Medicine, University of Pennsylvania Perelman School of Medicine, Philadelphia, PA USA; 6 Department of Physiology, Emory University School of Medicine, Atlanta, GA USA; 7 Neurological Disorders Research Center, Qatar Biomedical Research Institute (QBRI), Hamad Bin Khalifa University (HBKU), Education City, Qatar; 8 Life Sciences Division, College of Science and Engineering, Hamad Bin Khalifa University (HBKU), Education City, Qatar

## Correction

After publication of the original article [1] it was noted that the name of author, D. Luke Fisher, was erroneously typeset in both the PDF and online formats of the manuscript as Luke D. Fisher.

This error was introduced during typesetting, thus the publisher apologises for this error. The original article has been updated to reflect this correction.
