# Supplementary figures and images for: Lewy body-like alpha-synuclein inclusions trigger reactive microgliosis prior to nigral degeneration
Source: J Neuroinflammation. 2018 May 1;15:129. doi: 10.1186/s12974-018-1171-z (PMC5930695; doi:10.1186/s12974-018-1171-z)

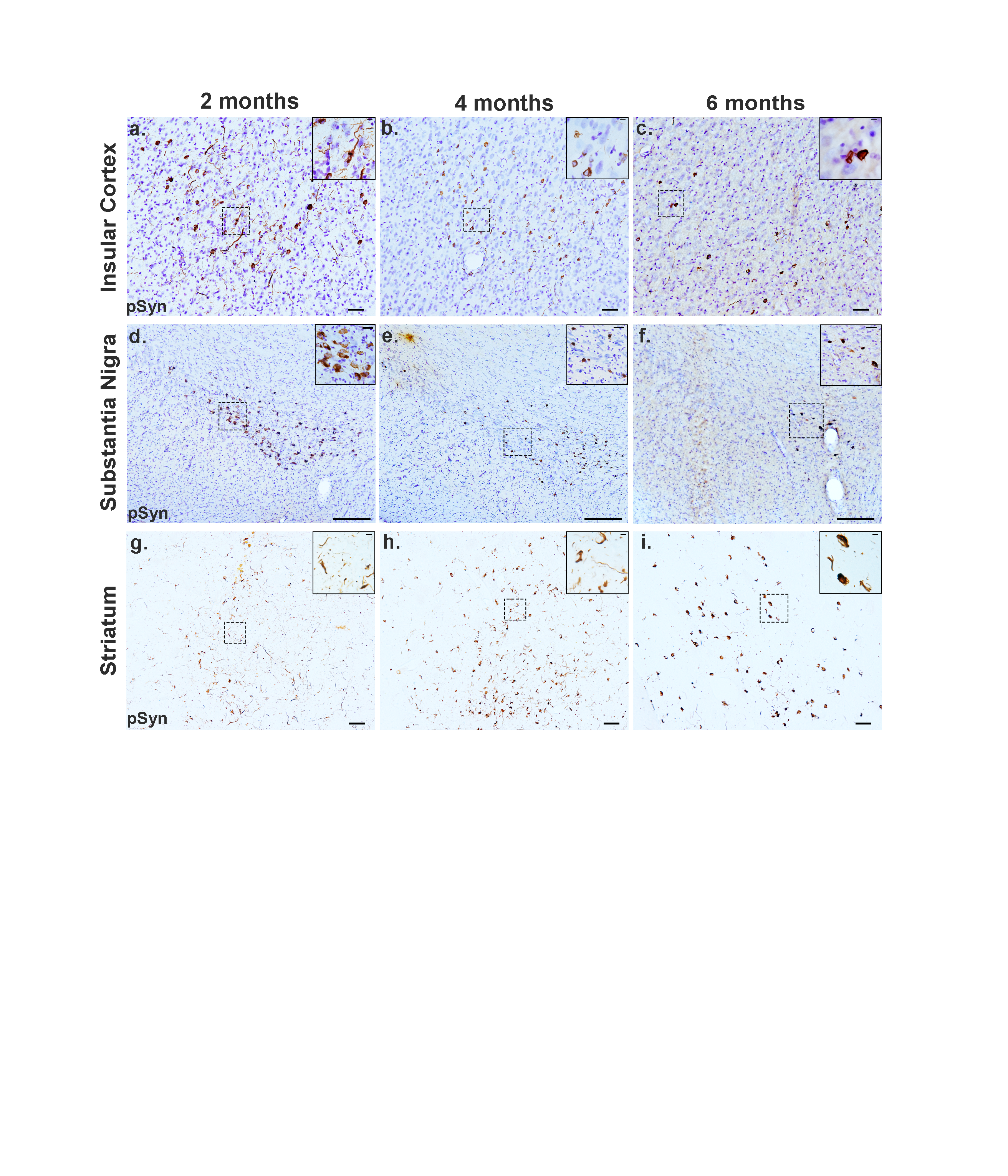

Supplement: Supplementary file 2 — Figure S2. Unilateral intrastriatal injection of α-syn PFFs induces widespread accumulation of Lewy-body like inclusions of phosphorylated α-syn (pSyn). Representative images illustrating the time course of pSyn pathology in regions innervating the striatum. (a–c) pSyn pathology in the ipsilateral agranular insular cortex localized to both the soma and neurites at 2 months p.i. (postinjection) that over time becomes primarily localized to the soma; scale bar = 50 μm, inset = 10 μm. (d–f) Ipsilateral accumulation of pSyn in the substantia nigra peaks at 2 months and becomes less abundant over time as neurons degenerate; scale bar = 200 μm, inset = 25 μm. (g–i) In contrast to other areas, pSyn in the striatum is primarily localized to neurites at 2 months and becomes more abundant and localized to the soma over time, scale bar = 50 μm, inset = 10 μm. Abbreviations: α-syn = alpha-synuclein; PFFs = pre-formed alpha-synuclein fibrils; pSyn = α-syn phosphorylated at serine 129; p.i. = postinjection. (TIF 33472 kb) [file 12974_2018_1171_MOESM2_ESM.tif]
